# Ceramide synthase 4 interferes with replication of influenza virus but is downregulated by infection

**DOI:** 10.1128/jvi.01563-25

**Published:** 2025-11-11

**Authors:** Kwang Il Jung, Chuan Xia, Savannah McKenna, Ying He, Vijayamahantesh Vijayamahantesh, Jennifer J. Wolf, Bumsuk Hahm

**Affiliations:** 1 Department of Surgery, University of Missouri 14716, Columbia, Missouri, USA; 2 Department of Molecular Microbiology and Immunology, University of Missouri 14716, Columbia, Missouri, USA; University Medical Center Freiburg, Freiburg, Germany

**Keywords:** influenza virus, ceramide synthase 4, JNK regulation, influenza-host interaction

## Abstract

Influenza continues to pose a serious threat to humans. Influenza-host interaction is incompletely understood, requiring identification of host factors that regulate viral pathogenicity. Ceramide synthases (CerSs) are responsible for producing and controlling ceramide levels within cells. Ceramides are structural and signaling sphingolipid components that mediate various biological functions and affect the infectivity of multiple viruses. However, the role of CerSs during virus infections remains unclear. In this study, we investigated the possible function of CerSs in host defense against influenza virus infection. Cells stably expressing CerS4 poorly supported influenza virus replication, whereas CerS1 did not affect replication. Transient overexpression of CerS4 also impaired the efficient production of viral proteins as well as infectious progeny viruses. In support of these results, knockdown of endogenous CerS4 in cells enhanced virus replication. Intriguingly, CerS4 impeded virus-induced activation of cellular c-Jun N-terminal kinase (JNK), which interfered with influenza viral replication. On the other hand, influenza virus infection was shown to induce CerS4 ubiquitination and downregulation, which could limit the antiviral activity of CerS4. Collectively, these findings reveal a new function of CerS4 that restricts influenza virus infection and provides valuable insights into influenza-host defense interactions.

**IMPORTANCE:**

Seasonal influenza causes serious public health problems in the world with substantial annual morbidity and mortality. Further, there have been persistent concerns about potential development of an influenza pandemic. Current antiviral drugs are limited in their efficacy, especially due to the rapid emergence of drug-resistant variants. Host protein-directed therapy is an alternative or complementary approach to broadly controlling influenza virus infections but requires a deeper understanding of influenza-host interplay. Host ceramide synthase 4 regulates the level of ceramides that possess both structural and signaling mediator functions. Our study reveals that ceramide synthase 4 displays an antiviral activity against influenza virus infection by regulating JNK activation. However, influenza virus triggers degradation of ceramide synthase 4, which could favor virus replication. The findings advance our knowledge about the ceramide network interaction with influenza and provide a framework for developing a host-targeted therapy to cure influenza.

## INTRODUCTION

Influenza remains a major public health concern, causing significant morbidity and mortality worldwide through both seasonal and pandemic influenza (1–4). The 2009 influenza pandemic, along with recurrent avian influenza outbreaks, has heightened the awareness about the risk of future influenza pandemics (5–9). Due to frequent genetic mutations of the influenza virus leading to antigenic variation, influenza vaccines must be updated yearly, and the vaccine’s efficacy varies year by year. Drugs that inhibit activity of viral proteins, such as NA, PA, and M2, have been used to mitigate the effects of influenza, but these treatments are constrained by the rise of drug-resistant strains (10–14). These drug-resistant viral variants, which include various seasonal and avian influenza viruses, undermine the effectiveness of current antiviral therapies (11, 13, 15). Therefore, there is an urgent need to design new therapeutics and complementary approaches, such as host-directed therapy, by identifying cellular targets to effectively control influenza virus infections. This requires a better understanding of influenza-host interaction and influenza viral pathogenesis.

Ceramide synthase (CerS) enzymes, which are integral membrane proteins found in the endoplasmic reticulum, consist of six distinct members designated as CerS1 through CerS6 (16–18). Each CerS enzyme has specificity to fatty acyl CoA chains of varying lengths and has differential tissue expression patterns (17, 19–21). The function of CerS has been studied in multiple disease conditions such as cancer, diabetes, and obesity, likely due to ceramide’s relevance to the regulation of cell death mechanisms or fatty acid metabolism (22–24). The inhibitor fumonisin B1, which blocks the activity of most of the CerS enzymes with limited specificity, was tested during influenza virus replication (25). However, the positive and negative results in separate studies yielded controversy (25–27), raising a question about the role of these enzymes upon influenza virus infection. CerS4, also known as LASS4 or TRH1, is the least studied CerS and has substrate specificity toward C18–C20 CoAs (19, 28, 29). It is primarily expressed in the lung and liver but also detected in multiple other tissues including the skin, nervous system, and pancreas (30). Unlike CerS1, CerS4 does not affect cellular sensitivity to chemotherapeutic drugs (31), but it is elevated in the brain of an Alzheimer’s disease mouse model (32). The function of CerS4 in viral infection has not been explored.

In this study, we investigated the possible role of CerSs, such as CerS4, during influenza virus replication. We found that CerS4 displays antiviral activity as it interferes with the replication and propagation of influenza viruses. The underlying mechanism involves the inhibition of JNK activation by CerS4. However, influenza virus induces a decrease of CerS4 at the protein level upon infection, suggesting the presence of a complex interplay between host CerS4 and influenza.

## RESULTS

### Stable overexpression of CerS4, but not CerS1, inhibits the replication of influenza virus

**Fig 1:**
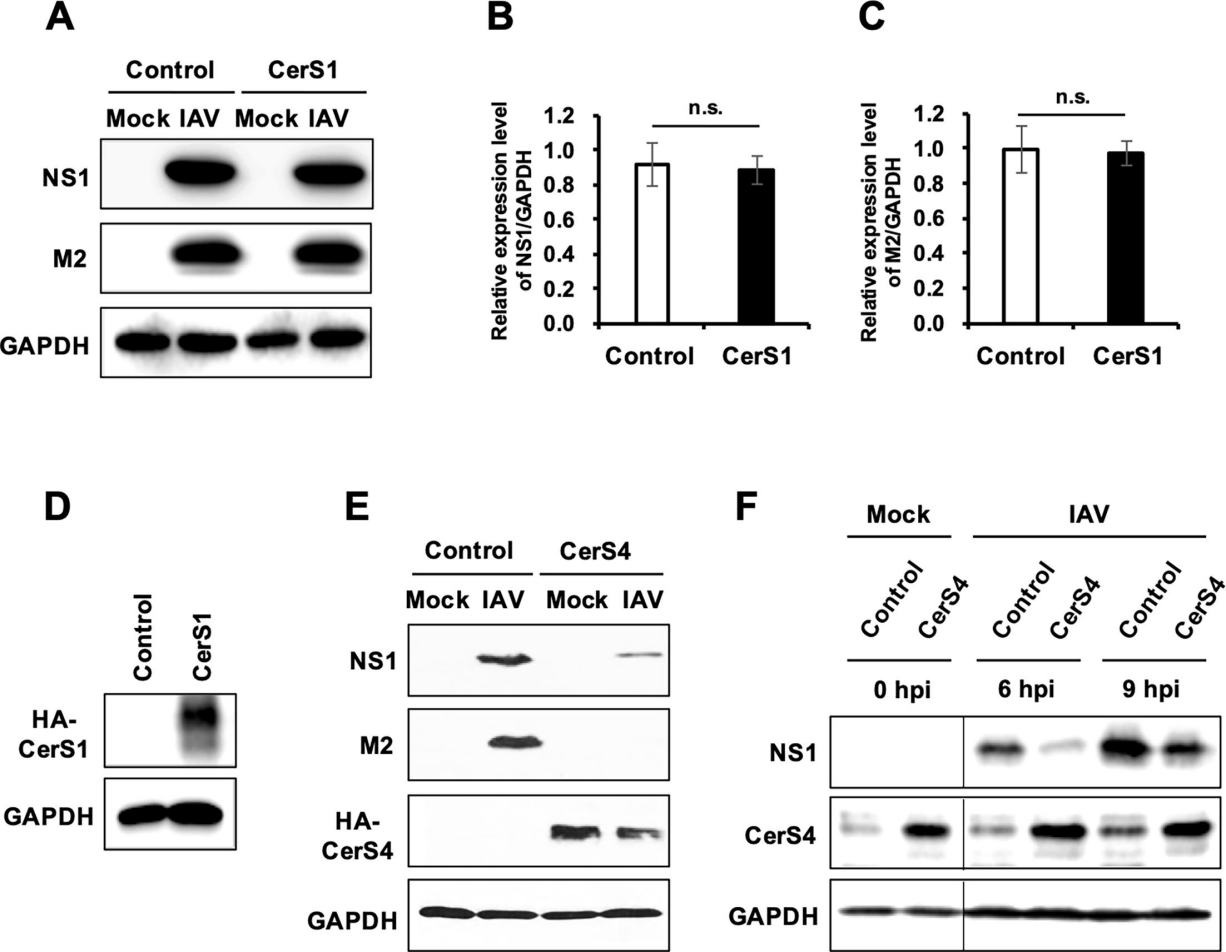
Stable overexpression of CerS4, but not CerS1, inhibits the replication of IAV. (**A**) HEK293 cells (Control) or HEK293 cells overexpressing HA-tagged CerS1 (CerS1) were either left uninfected (Mock) or infected with IAV (pH1N1) at an MOI of 1. At 24 hours post-infection (hpi), the cells were harvested, and Western blotting was performed to detect the levels of viral NS1, viral M2, and GAPDH. (**B and C**) Densitometric analysis of NS1/GAPDH (**B**) and M2/GAPDH (**C**) from three replicates of pH1N1-infected Control vs. CerS1, as shown in panel (**A**). (**D**) Western blot analysis was conducted to detect HA-tagged CerS1 in HEK293 cells overexpressing CerS1. (**E**) HEK293 cells (Control) or HEK293 cells overexpressing HA-tagged CerS4 (CerS4) were infected with IAV (WSN) at an MOI of 0.1. At 24 hpi, cells were harvested, and levels of viral NS1, viral M2, HA-tagged CerS4 (HA-CerS4), and GAPDH were detected by immunoblotting. (**F**) Control HEK293 cells or HEK293 cells overexpressing HA-tagged CerS4 (CerS4) were either left uninfected (Mock) or infected with IAV (pH1N1) at an MOI of 1. At 0, 6, and 9 hpi, the cells were harvested, and Western blotting was performed to detect the levels of viral NS1, CerS4, and GAPDH. In all experiments, GAPDH levels were measured to serve as an internal loading control. n.s., not significant.

Previously, CerS1 and CerS4 were studied using cells stably overexpressing each enzyme with the treatment of anticancer agents to determine their functions during the stress response (31). In this study, these cells were used to determine the possible roles of CerS1 and CerS4 during influenza virus infection. Since CerS1, but not CerS4, regulated the cell death induced by the anticancer agent cisplatin, we initially tested CerS1 by comparing control and CerS1-overexpressing HEK cells in terms of cellular susceptibility to IAV infection. However, stable overexpression of CerS1 did not affect the expressions of viral proteins such as NS1 and M2 of 2009 pandemic influenza A/CA/04/09 (H1N1) (pH1N1) in Western blot analysis (Fig. 1A through D). Interestingly, stable overexpression of CerS4 inhibited the expressions of viral proteins upon IAV infection (Fig. 1E and F). The inhibition of viral protein expression by CerS4 was observed upon influenza A/WSN/33 (H1N1) (IAV WSN) (Fig. 1E) or pH1N1 at multiple time points after infection (Fig. 1F). As IAV WSN replicates more rapidly than pH1N1 in the culture condition, a lower multiplicity of infection (MOI) (MOI = 0.1) was used for infection with WSN than pH1N1 (MOI = 1.0). The results indicate that stable expression of CerS4 impairs IAV replication.

### Transient overexpression of CerS4 impairs replication and propagation of influenza viruses

**Fig 2:**
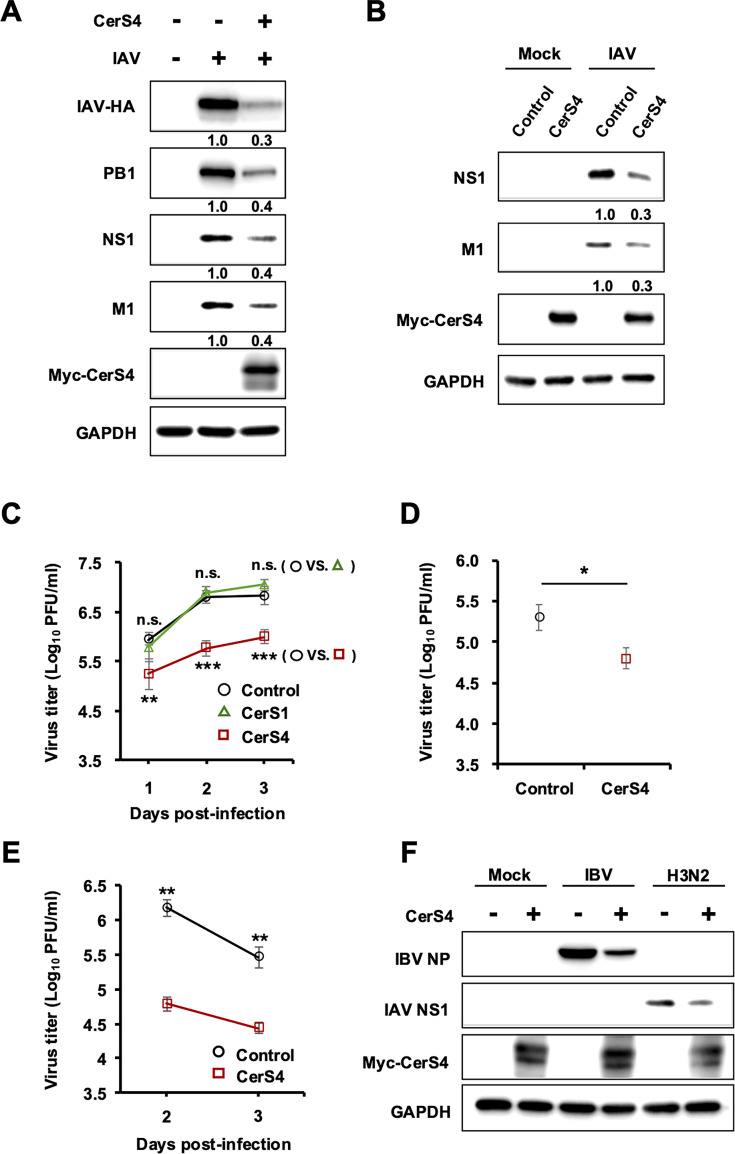
Transient overexpression of CerS4 impairs productive infection of influenza viruses. (**A**) HEK293FT cells were transfected with control plasmid (−) or plasmid encoding Myc-tagged CerS4 (+). The cells were mock-infected or infected with IAV (pH1N1) at an MOI of 1 at 18  hours post-transfection. At 24 hpi, cells were harvested, and Western blotting was performed to detect the levels of IAV-HA, PB1, NS1, M1, Myc-tagged CerS4, and GAPDH. Densitometric values are shown, with relative expression of viral protein/GAPDH in vector control and infected samples set to 1.0. The effect of CerS4 overexpression in infected cells was compared. (**B**) A549 cells were transfected with vector control or CerS4-encoding plasmid. At 18 hours after transfections, cells were mock-infected or infected with pH1N1 at an MOI of 3 for 8 hours. Expression levels of viral proteins were assessed as shown in panel (**A**). (**C–E**) A549 cells were transfected with a vector control plasmid (Control) or plasmid encoding CerS4 (CerS4) or plasmid encoding CerS1 (CerS1, Fig. 2C only). In (**C**), at 18 hours post-transfection, the cells were infected with IAV pH1N1 at an MOI of 0.01. At 1 (*n* = 7/group), 2 (*n* = 3/group), or 3 (*n* = 6/group) dpi, cellular supernatants were harvested. Control vs CerS1 and Control vs CerS4 titers were statistically analyzed as indicated. In (**D**), A549 cells were infected with IAV pH1N1 at an MOI of 3 for 8 hours, followed by supernatant collection for titration by plaque assay (*n* = 3/group). In (**E**), A549 cells were infected with IAV WSN at an MOI of 0.001. Cell supernatants were collected at 2 and 3 dpi for assessing viral titers using plaque assay on MDCK cells (*n* = 3/group). Statistical significance was determined by a *t*-test and is indicated by * *P* < 0.05, ** *P* < 0.01, and *** *P* < 0.001. Data are expressed as means ± SD. (**F**) HEK293FT cells were transfected with control plasmid (−) or plasmid encoding Myc-tagged CerS4 (+) and were infected with influenza B/Lee/40 virus (IBV) or A/Hong Kong/8/68 (H3N2) at an MOI of 1 at 18  hours post-transfection. At 24 hpi, cells were harvested, and Western blotting was performed to detect the levels of IBV NP, IAV NS1, Myc-tagged CerS4, and GAPDH. The data are representative of three independent experiments.

We next determined if transient overexpression of CerS4 regulates influenza virus replication similar to the stable overexpression of CerS4. To this end, cells were transfected with CerS4-encoding plasmid or vector control DNA and then infected with pH1N1 at an MOI of 1.0 for 24 hours (h). Transiently overexpressed CerS4 interfered with the replication of IAV, as evidenced by the reduced expression levels of many viral proteins detected by immunoblotting (Fig. 2A). The inhibition of viral protein expression by CerS4 was also observed when cells were infected with pH1N1 at an MOI of 3.0 for 8 h, representing a single-cycle infection condition (Fig. 2B). Importantly, CerS4 overexpression substantially decreased the production of infectious influenza virus particles from IAV-infected human lung epithelial A549 cells compared to the control in multiple experimental conditions, as measured by plaque assay (Fig. 2C through E): CerS4, but not CerS1, interfered with the production of infectious viruses at 1, 2, and 3 dpi when cells were infected with pH1N1 at an MOI of 0.01 (Fig. 2C). Further, CerS4 repressed the generation of infectious virus progeny when cells were infected with pH1N1 at an MOI of 3.0 for 8 h (Fig. 2D) or infected with IAV WSN strain at an MOI of 0.001 (Fig. 2E). To determine whether the effects of CerS4 on influenza are limited to IAV, influenza B/Lee/40 virus (IBV) and influenza A/Hong Kong/8/68 (H3N2) virus (IAV H3N2) were also examined (Fig. 2F). The overexpression of CerS4 led to the inhibition of viral protein expression when cells were infected by IBV or IAV H3N2 (Fig. 2F), suggesting that CerS4 regulates the replication of multiple influenza virus types and subtypes. Taken together, these data indicate that overexpressed CerS4 displays antiviral function and impairs the productive infection of influenza viruses.

### Knockdown of endogenous CerS4 enhances the replication of influenza virus

**Fig 3:**
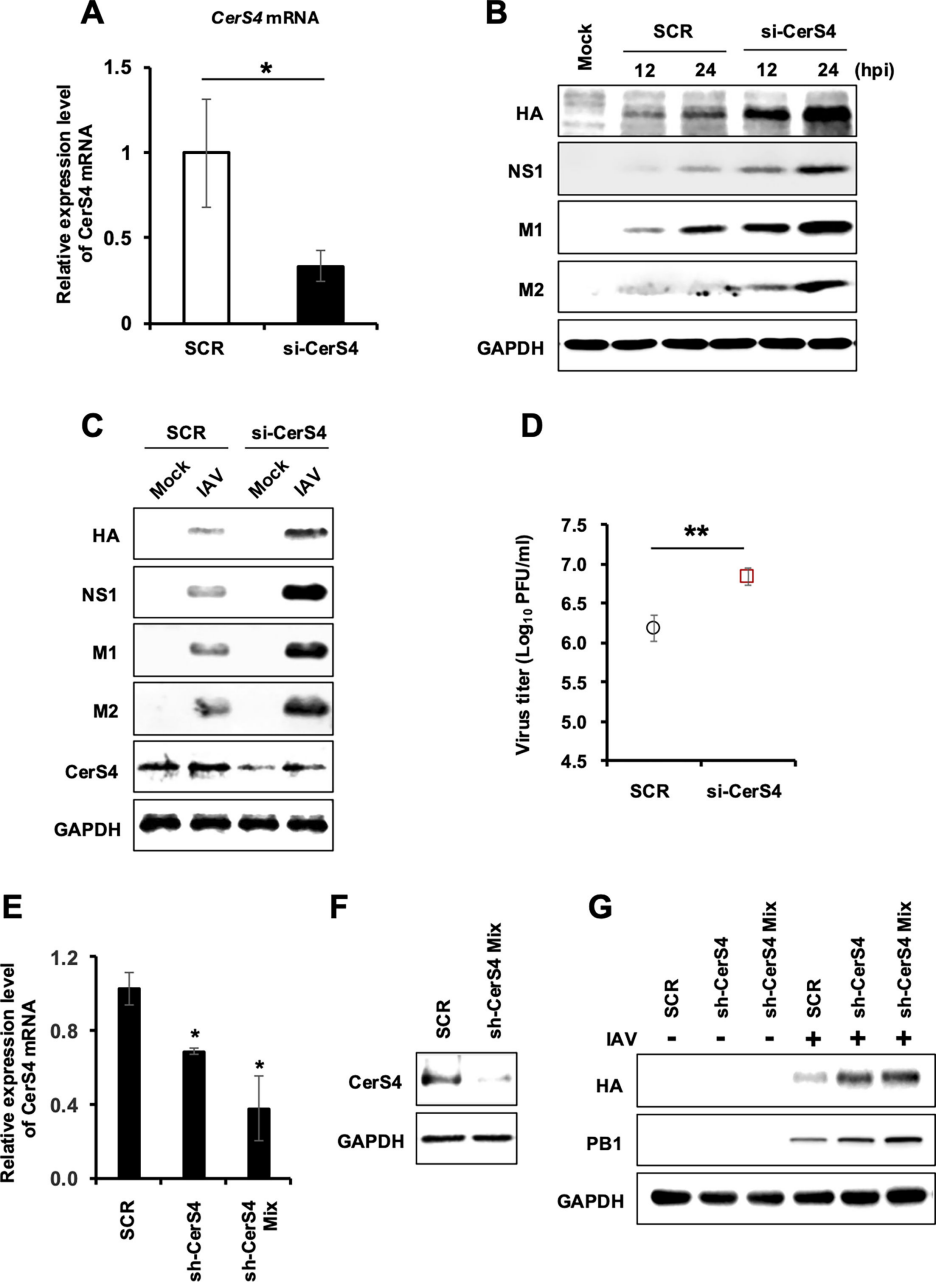
Knockdown of endogenous CerS4 increases IAV replication. (**A**) HEK293T cells were transfected with a control scrambled RNA (SCR) or CerS4-specific siRNA (si-CerS4). The mRNA levels of CerS4 were analyzed by real-time qPCR at 18 hours post-transfection (*n* = 3/group). (**B**) HEK293T cells were transfected with SCR or si-CerS4. At 18 hours after transfection, cells were mock-infected or infected with IAV (pH1N1) at an MOI of 0.1 for 12 or 24  hours. Western blotting was performed to detect the levels of viral HA, NS1, M1, and M2 along with GAPDH. (**C**) HEK293T cells were transfected with SCR or si-CerS4. At 18 hours post-transfection, cells were uninfected (mock) or infected with IAV (WSN) at an MOI of 0.1 for 24  hours. Western blotting was performed to detect the levels of viral HA, NS1, M1, and M2, CerS4, as well as GAPDH. (**D**) A549 cells were transfected with SCR or si-CerS4 and infected with IAV (WSN) at an MOI of 0.001 at 18 hours post-transfection. At 2 dpi, viral progeny produced from the cells were quantified using plaque assay on MDCK cells. Viral production is shown as PFU per mL. Data are expressed as means ± SD (*n* = 3/group). (**E–G**) A549 cells were transduced with sh-SCR-encoding control lentivirus (SCR) or CerS4-specific shRNA (sh-CerS4) encoding lentiviruses (sh-CerS4 or sh-CerS4 Mix that has two types of shRNAs). (**E**) At 72 hours after transduction, mRNA levels of CerS4 were measured by real-time qPCR. (**F**) At 72 hours after transduction, endogenous CerS4 was detected by Western blotting. (**G**) After 48 hours, cells were mock-infected or infected with IAV (pH1N1) at an MOI of 0.1 for 24  hours. Western blotting was performed to detect the levels of viral PB1, viral HA, and GAPDH. Statistical significance was determined by a *t*-test and is indicated by *P < 0.05 and ** *P* < 0.01. Data are expressed as means ± SD (*n* = 3/group). The experiment was repeated with similar results.

To investigate the role of endogenous CerS4 during influenza virus infection, we implemented a knockdown method utilizing small interfering RNA (siRNA) designed to specifically target CerS4 (si-CerS4). Following the downregulation of CerS4 with si-CerS4, an increase in viral protein expression levels was observed at both 12 and 24 hours post-infection (hpi) with IAV pH1N1 compared to scrambled siRNA control (SCR) (Fig. 3A and B). Similarly, viral protein expression was elevated in cells infected with the IAV WSN strain when CerS4 expression was knocked down (Fig. 3C). Furthermore, downregulation of CerS4 using siRNA resulted in increased production of infectious IAV particles, which was assessed by plaque assay (Fig. 3D).

To confirm the antiviral role of endogenous CerS4 during influenza virus infection, recombinant lentiviruses were constructed to deliver shRNA targeting CerS4 (sh-CerS4) and inhibit the expression of endogenous CerS4. A549 cells were transduced with lentivirus encoding sh-CerS4, a mixture of multiple sh-CerS4 (sh-CerS4 Mix) or control lentivirus, and then infected with IAV to assess the impact of CerS4 downregulation on the level of viral replication. sh-CerS4-mediated CerS4 downregulation (Fig. 3E and F) led to an increase in the expressions of viral proteins HA and PB1 (Fig. 3G). The results further demonstrate that endogenous CerS4 acts as an antiviral host factor during influenza virus infection.

### CerS4 inhibits JNK activation, which is associated with CerS4’s antiviral activity during infection

**Fig 4:**
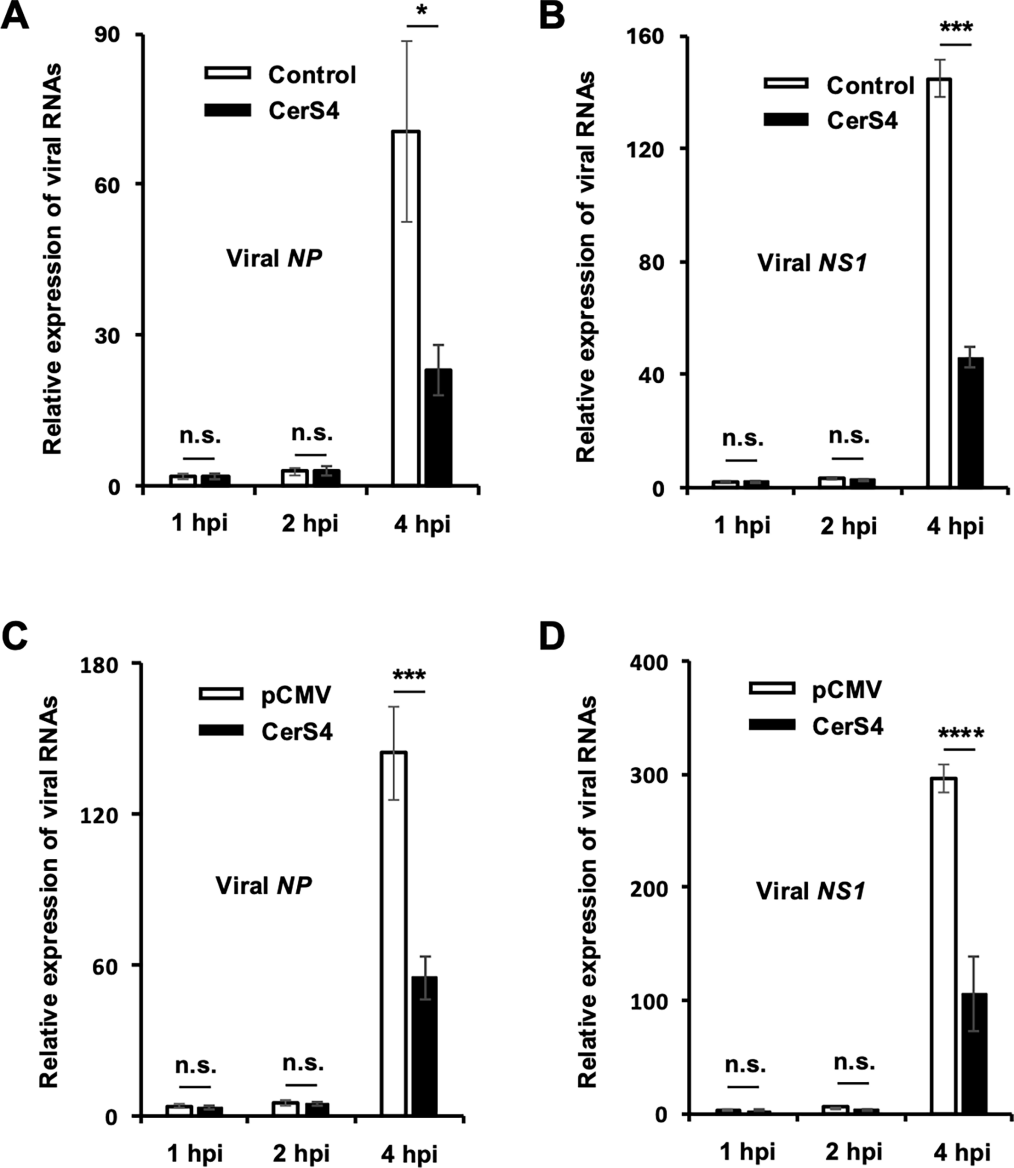
Overexpression of CerS4 inhibits the expression of viral RNAs over time following influenza virus infection. (**A and B**) HEK293 control cells (Control) or HEK293 cells overexpressing CerS4 (CerS4) were infected with IAV (pH1N1) at an MOI of 3. (**C and D**) A549 cells were transfected with a control vector plasmid (pCMV) or CerS4-encoding plasmid (CerS4), and at 18 hpi, they were infected with IAV (pH1N1) at an MOI of 3. (**A–D**) At 1, 2, and 4 hpi, the cells were harvested (*n* = 3/group). Reverse transcription was performed with primers complementary to viral (−) strand RNA. Levels of NP and NS1-specific (−) strand viral RNAs were measured by real-time qPCR, with mRNA for GAPDH serving as the internal reference. In each panel, the viral gene/GAPDH level in control cells at 1 hpi was set to 1.0. Statistical significance was determined by a *t*-test and is indicated by n.s., not significant, * *P* < 0.05, *** *P* < 0.001 and **** *P* < 0.0001. Data are expressed as means ± SD (*n* = 3/group). The experiment was repeated with similar results.

The main stages of the influenza virus life cycle include viral entry, viral replication, and the release of virus particles from the host cell (33, 34). To further understand the mechanism of CerS4 inhibition of IAV replication, we conducted reverse transcription (RT) with viral (−) strand RNA-complementary primers followed by real-time qPCR using IAV-infected CerS4-overexpressing cells and control cells. The synthesis of viral (−) strand RNAs specific for NP (Fig. 4A) and NS1 (Fig. 4B) was inhibited at 4 hpi in CerS4-overexpressing cells compared to control cells. However, no changes in the levels of viral (−) strand RNA were observed between control and CerS4- overexpressing cells at 1 hpi or 2 hpi (Fig. 4A and B). A similar pattern of viral RNA regulation was detected when CerS4 was transiently overexpressed in A549 cells followed by pH1N1 infection (Fig. 4C and D). The results imply that the inhibition of viral replication by CerS4 did not occur at very early steps of viral infection such as viral entry.

**Fig 5:**
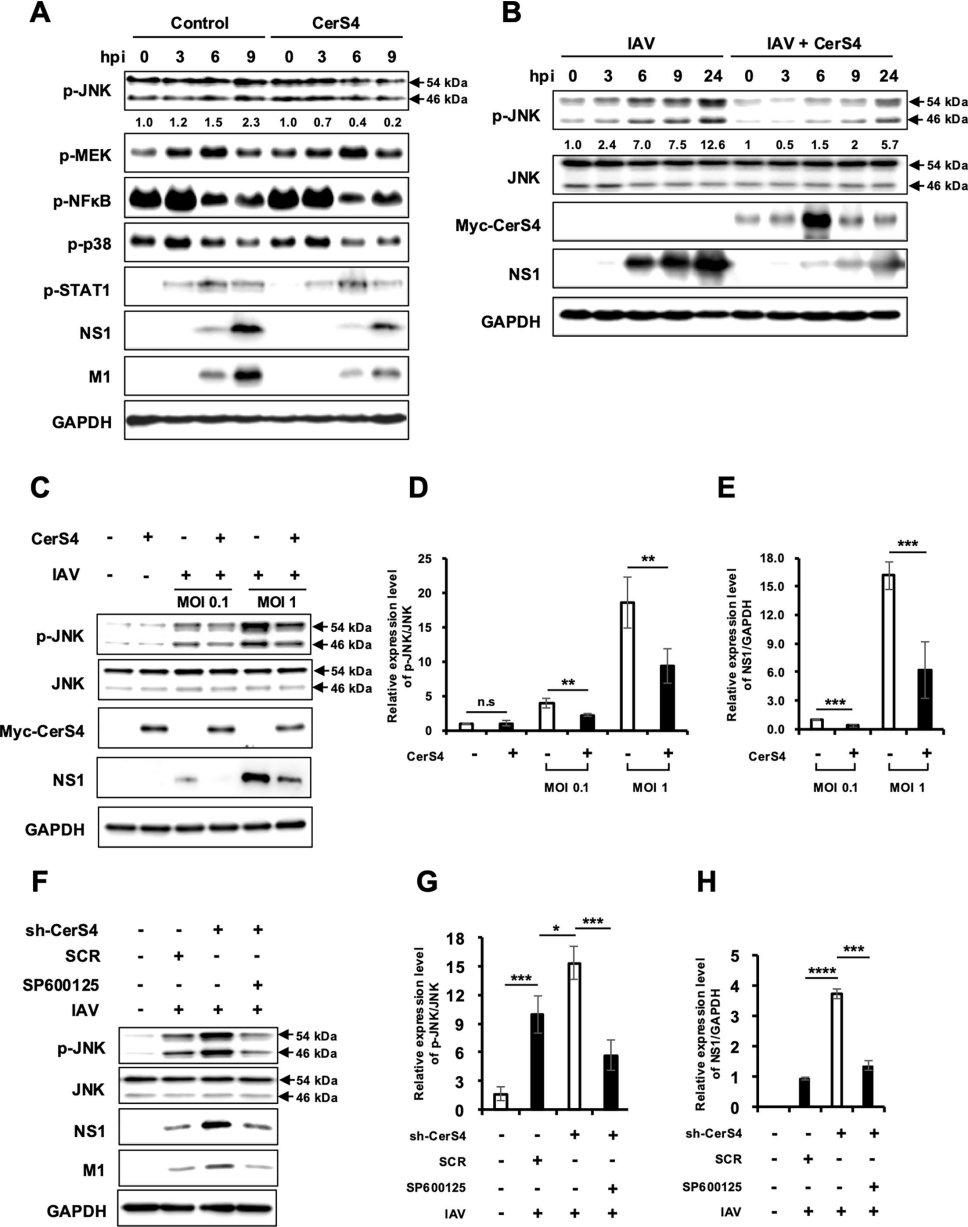
CerS4 inhibits JNK activation, which is associated with impaired replication of influenza virus. (**A**) HEK293 cells (Control) or HEK293 cells stably overexpressing CerS4 (CerS4) were mock-infected or infected with IAV (pH1N1) at an MOI of 1. After 0, 3, 6, and 9 hpi, the cells were harvested. Western blotting was performed to detect the levels of p-JNK, p-MEK, p-NF-κB, p-p38 MAPK, p-STAT1, viral NS1, viral M1, and GAPDH. The data are representative of three independent experiments. (**B**) HEK293 cells were transfected with an empty vector or plasmids encoding Myc-tagged CerS4. At 24 hours post-transfection, cells were infected with IAV (WSN) at an MOI of 0.01. At 0, 3, 6, 9, and 24 hpi, cells were harvested, and Western blotting was performed to detect the levels of p-JNK, total JNK, Myc-tagged CerS4, viral NS1, and GAPDH. (**C–E**) A549 cells were transfected with an empty vector or plasmids encoding Myc-tagged CerS4. At 24 hours post-transfection, cells were mock-infected or infected with IAV (pH1N1) at an MOI of 0.1 or 1.0. At 24 hpi, cells were harvested, and Western blotting was performed to detect the levels of p-JNK, total JNK, Myc-tagged CerS4, viral NS1, and GAPDH. (**D**) Relative expression of p-JNK/JNK from panel (**C**) was quantitated by densitometric analysis. Expression in control vector and mock-infected cells was set to 1.0. (*n* = 4/group). (**E**) Relative expression of viral NS1/GAPDH from panel (**C**) was quantitated by densitometric analysis. Expression in control vector and MOI 0.1-infected cells was set to 1.0. (*n* = 4/group). (**F–H**) A549 cells were transduced with SCR or sh-CerS4 encoding lentivirus. At 48 hours post-transduction, the cells were mock-infected or infected with IAV (pH1N1) at an MOI of 0.1. At 7 hours prior to cell harvesting, the cells were treated with either solvent control (DMSO) or 20 µM SP600125. At 24 hpi, the cells were harvested, and Western blotting was performed to detect the levels of p-JNK, total JNK, viral NS1, viral M1, Myc-tagged CerS4, and GAPDH. (**G**) Relative expression of p-JNK/JNK from panel (**F**) was quantitated by densitometric analysis. Expression in Lane 1 cells was set to 1.0. (*n* = 3/group). (**H**) Relative expression of viral NS1/GAPDH from panel (**F**) was quantitated by densitometric analysis. Expression in Lane 2 cells was set to 1.0. The arrows indicate two forms of p-JNK or JNK (46 kDa and 54 kDa). (*n* = 3/group). n.s., nonsignificant, * *P* < 0.05, ** *P* < 0.01, *** *P* < 0.001, and **** *P* < 0.0001.

While it is unknown whether CerS4 regulates specific cellular signaling pathways, ceramide has been reported to activate multiple cellular signaling proteins depending on the disease condition (35–37). To investigate the underlying mechanism by which CerS4 regulates IAV replication, the activations of several signaling components important for IAV replication or host defense were monitored in control cells and CerS4-overexpressing cells at 0, 3, 6, and 9 hours after IAV infection (Fig. 5A). When viral proteins were detected at 6 and 9 hpi, correlative decreases in phosphorylated forms of JNK (p-JNK) were observed in CerS4-overexpressing cells compared to those in control cells. However, the activation status of MEK, NF-κB, STAT1, and p38 MAPK (pMEK, pNF-κB, pSTAT1, and p-p38) remained largely unchanged by CerS4 overexpression during IAV infection. Since JNK inhibition was reported to repress IAV replication (38, 39), the finding led us to hypothesize that CerS4 regulates JNK activation and impairs viral replication.

Next, we determined if transient overexpression of CerS4 affects the phosphorylation of JNK during IAV replication. Infection by IAV WSN increased the levels of p-JNK. Transient overexpression of CerS4 did not affect the levels of total JNK proteins but impaired the increase in p-JNK during WSN infection over time, which was associated with the inhibition of viral protein expression (Fig. 5B). The inhibition of p-JNK levels by transiently overexpressed CerS4 during infection was also observed when A549 cells were infected by pH1N1 at an MOI of 0.1 and 1.0 (Fig. 5C through E), where virus-induced increase of p-JNK was more evidently observed at an MOI of 1.0, presumably due to the higher MOI used for infection. Since immunoblotting can detect a one-time measurement, we used two different experimental conditions (MOI of 0.1 and 1.0) to find that CerS4 mediates the inhibition of p-JNK in multiple experimental conditions. Further, when the endogenous CerS4 was downregulated using sh-CerS4 encoding lentivirus, the levels of IAV-induced p-JNK, as well as viral protein expression, increased (Fig. 5F through H). To further support the result, JNK activation was blocked by the treatment with SP600125, a specific JNK inhibitor. In the presence of the JNK inhibitor, the effects of CerS4 downregulation on both p-JNK and viral protein expressions were abrogated (Fig. 5F through H), suggesting that CerS4’s regulation of JNK is important for CerS4-mediated suppression of IAV replication.

**Fig 6:**
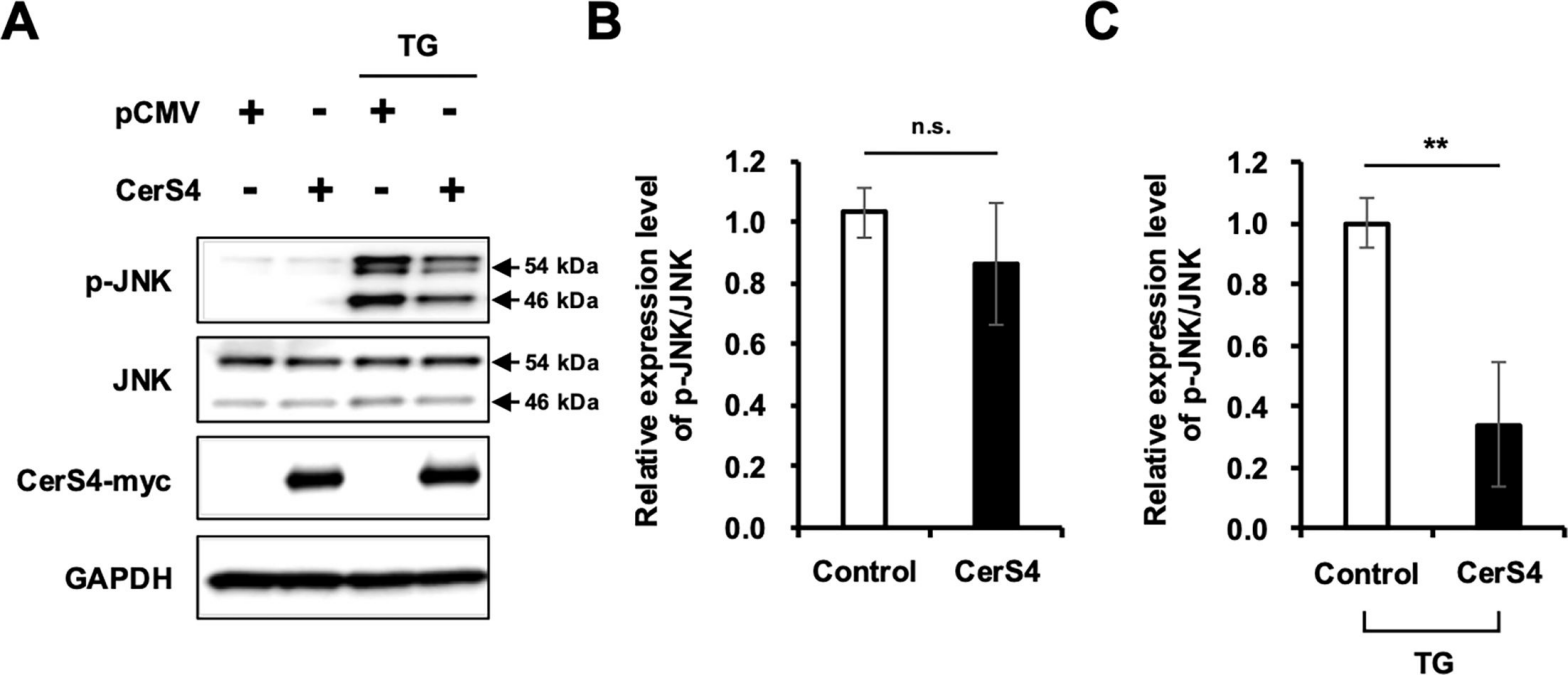
CerS4 inhibits JNK phosphorylation in cells treated with thapsigargin. A549 cells were transfected with pCMV control vector or CerS4-encoding plasmid. At 40 hours post-transfection, cells were treated with either solvent control (DMSO) or 1 mM thapsigargin (TG) for 2 hours. (**A**) Cells were assessed for expression of p-JNK and JNK by immunoblotting. The arrows indicate two forms of p-JNK or JNK (46 kDa and 54 kDa). (**B**) Relative expression of p-JNK/JNK from cells transfected with control vector or CerS4-encoding plasmid without TG treatment from panel (**A**) was quantitated by densitometric analysis (*n* = 3/group). (**C**) Relative expression of p-JNK/JNK from cells transfected with control or CerS4-encoding plasmid with TG treatment from panel (**A**) was quantitated by densitometric analysis (*n* = 3/group). n.s. = nonsignificant, ** *P* < 0.01.

Although our experiments were performed with kinetic monitoring of signaling components and incorporation of JNK inhibitor, it may be difficult to fully exclude the possibility that CerS4 inhibits IAV replication via an as-yet undefined mechanism, which leads to suppression of IAV-induced JNK activation. Indeed, CerS4 or ceramide-mediated inhibition of p-JNK has not been reported. Therefore, we determined whether CerS4 regulates p-JNK under another condition in the absence of viruses. For this purpose, A549 cells were stimulated with thapsigargin (TG), which is known to activate JNK. CerS4 overexpression was demonstrated to inhibit TG-induced JNK activation (Fig. 6A through C). The results indicate that CerS4 can inhibit JNK activation in multiple conditions.

### Influenza virus infection induces degradation of CerS4

**Fig 7:**
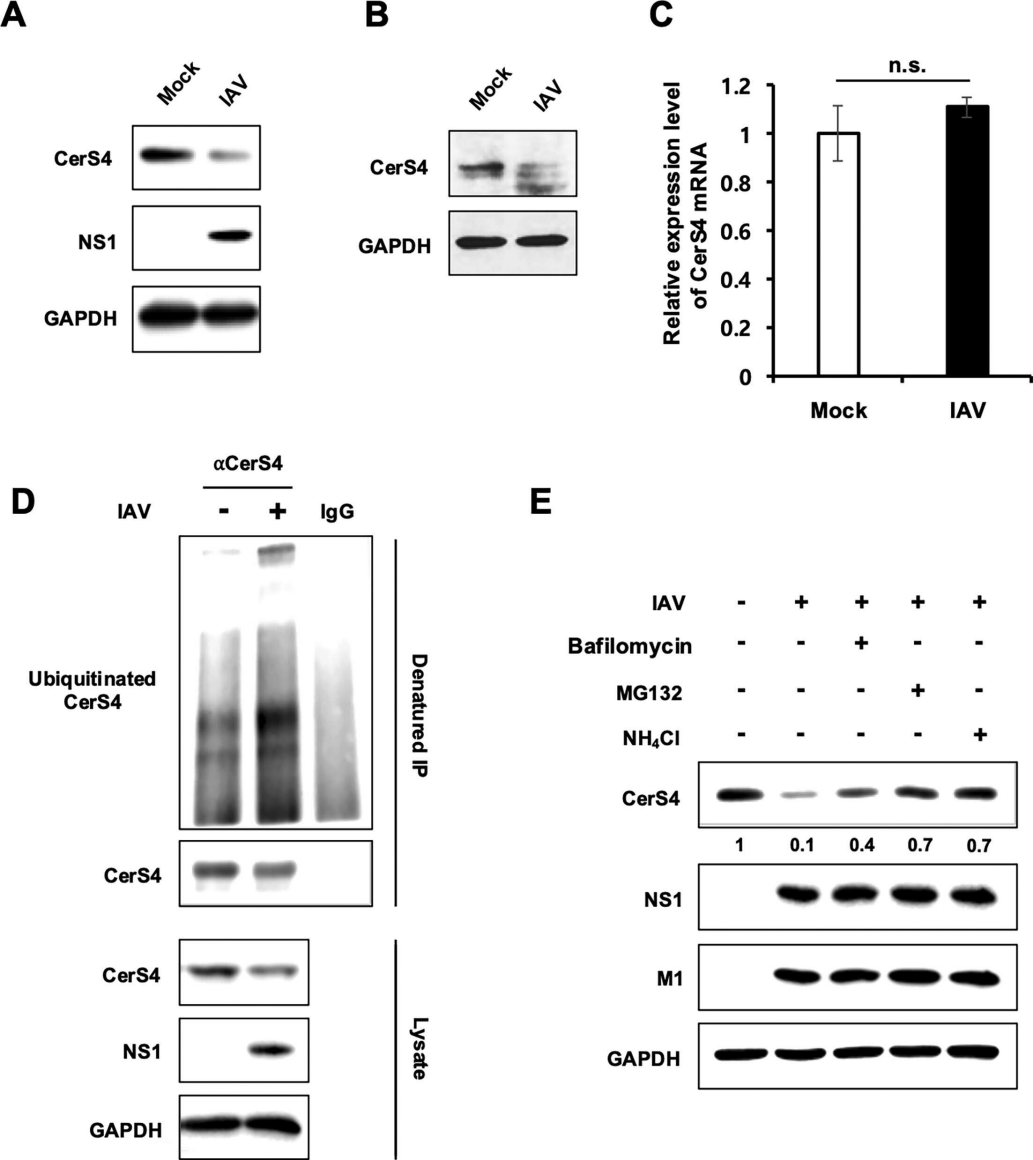
Influenza virus infection induces degradation of CerS4. (**A**) A549 cells were mock-infected or infected with IAV (pH1N1) at an MOI of 1. At 24 hpi, Western blot analysis was conducted to detect the levels of CerS4, viral NS1, and GAPDH. (**B**) HEK293 cells were mock-infected or infected with IAV (WSN) at an MOI of 1. At 12 hpi, Western blotting was performed to detect the levels of CerS4 and GAPDH. (**C**) A549 cells were mock-infected or infected with IAV (pH1N1) at an MOI of 1. At 24 hpi, RNA was extracted. CerS4 mRNA levels were measured by real-time qPCR. Statistical significance was determined by *t*-test and is indicated by n.s., nonsignificant. Data are expressed as means ± SD of three separate experiments. (**D**) A549 cells were mock-infected or infected with IAV (pH1N1) at an MOI of 2. At 24 hpi, the cells were harvested. Immunoprecipitation of total lysates was performed using an anti-CerS4 antibody (αCerS4), followed by Western blot detection of ubiquitin, CerS4, viral NS1, and GAPDH. IgG antibody was used for immunoprecipitation as a control. Ubiquitinated CerS4 was separated on a 10% SDS-PAGE. (**E**) A549 cells were mock-infected or infected with IAV (pH1N1) at an MOI of 1. At 18 hpi, cells were treated with 50 nM bafilomycin A1, 40 µM MG132, or 40 mM NH_4_Cl for the final 6 hours before harvest. At 24 hpi, the cells were harvested, and Western blotting was performed to detect the levels of CerS4, viral NS1, viral M1, and GAPDH. The data are representative of three independent experiments.

While studying the role of CerS4 during IAV replication, we observed that IAV infection induced downregulation of endogenous CerS4 in multiple cell types including A549 (Fig. 7A) and HEK293 cells (Fig. 7B). However, the levels of mRNA encoding CerS4 did not significantly decrease upon IAV infection (Fig. 7C), suggesting that CerS4 regulation takes place at the post-transcriptional level during infection. These observations led us to hypothesize that IAV infection induces degradation of CerS4 protein, which could be beneficial for IAV replication. To determine if IAV infection triggers ubiquitination of CerS4, endogenous CerS4 was immunoprecipitated using anti-CerS4 antibody followed by the detection of ubiquitinated forms of CerS4 by Western blotting. Indeed, IAV infection increased the level of ubiquitinated CerS4 protein in A549 cells (Fig. 7D). To further evaluate CerS4 degradation during infection, we used inhibitors that block proteasomal (MG132) or lysosomal (NH_4_Cl and Bafilomycin A1) protein degradation pathways. Cells were infected with influenza virus and only treated with inhibitor for 6 hours prior to harvest as earlier treatment could affect IAV replication, which would subsequently impair virus-induced CerS4 degradation. A549 cells treated with any of the inhibitors (MG132, NH_4_Cl, or bafilomycin A1) inhibited IAV-induced CerS4 downregulation (Fig. 7E). The results corroborate the conclusion that IAV induces degradation of CerS4.

## DISCUSSION

**Fig 8:**
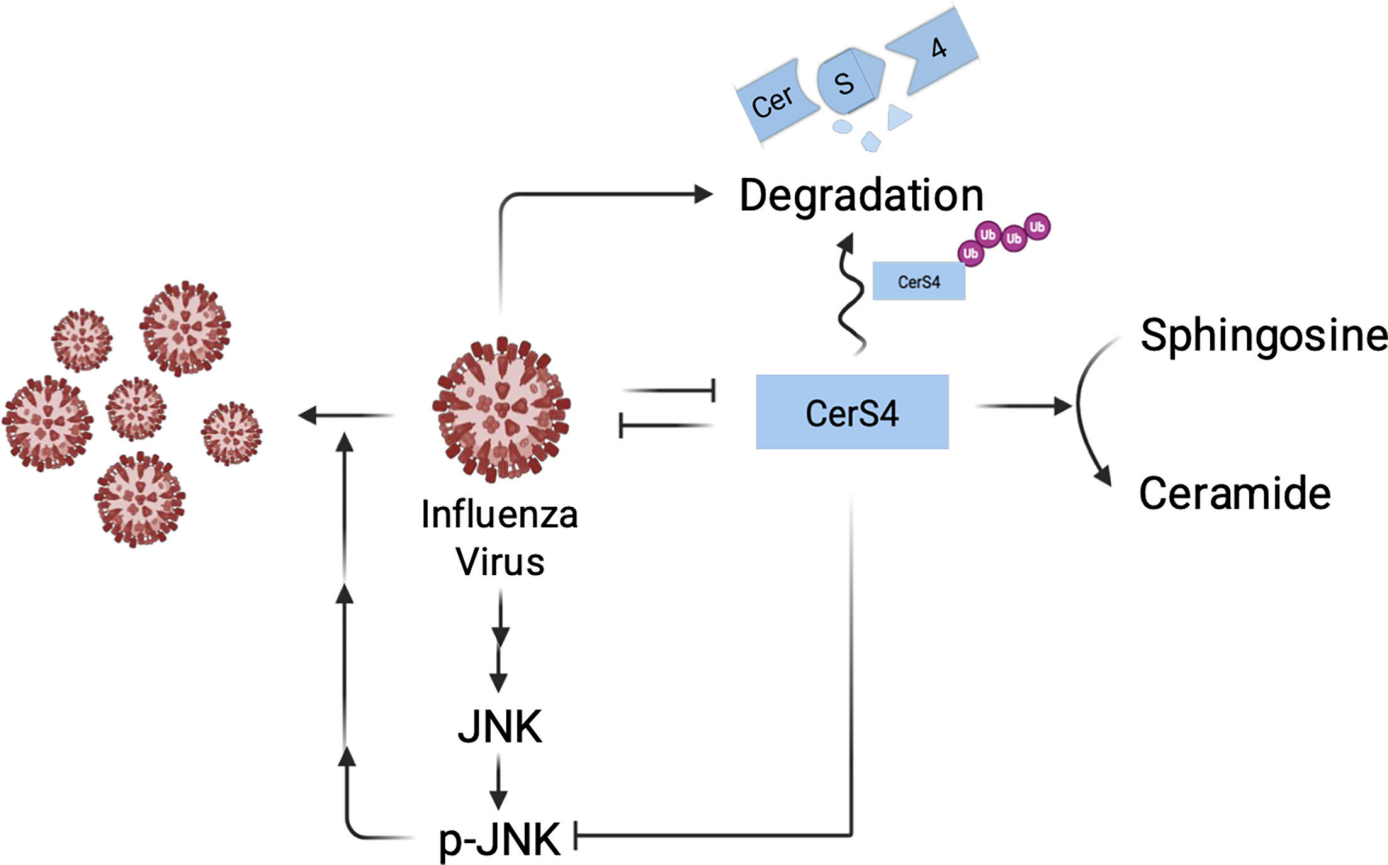
Model for the interaction between influenza virus and CerS4. CerS4 inhibits influenza virus-induced phosphorylation of JNK, which is known to be important for effective replication of influenza virus. On the other hand, influenza virus infection triggers the ubiquitination and subsequent degradation of CerS4, which could restrict the antiviral function of CerS4.

Using multiple genetic modification methods of stable or transient overexpression of CerS4 as well as CerS4 knockdown by siRNA or shRNA, this study revealed that CerS4 acts as an antiviral protein during influenza virus infection. However, influenza virus may strive to counteract the antiviral activity of CerS4 as infection triggers degradation of CerS4, which in turn could promote a cellular environment advantageous for the replication of influenza viruses (Fig. 8).

In prior cancer research, CerS1, but not CerS4, increased cellular sensitivity to cisplatin, a chemotherapeutic drug, with enhanced cell death. Activation of p38 MAPK, which is a well-known stress kinase, is increased by CerS1, but not by CerS4 in response to cisplatin treatment, explaining the enhanced cell death by CerS1 (31). However, upon influenza virus infection, p38 MAPK was not activated by CerS4. The difference in activation of signaling components by various stress stimuli or pathogen may explain the involvement of specific CerS under distinct disease conditions. Ceramide has been reported to increase cell stress responses and activate multiple signaling proteins, including ASK1, p38 MAPK, and JNK (40–43). Therefore, the inhibition rather than activation of JNK by CerS4 was surprising. The inhibition of JNK activation by CerS4 was also detected when cells were stimulated by TG. Thus, the results suggest that JNK inhibition may represent a CerS4-specific regulatory mechanism. It is currently unknown if CerS4 or the metabolic product, such as C20 ceramide, directly regulates JNK activation. As JNK can be activated by multiple signaling pathways that include upstream kinases (44), it is also possible that CerS4 targets a specific signaling molecule upstream of JNK. The regulatory mechanism could be common to the pathway activated by TG and influenza virus infection (45). The detailed mechanism remains to be explored.

The role of ceramide during virus infections has been previously investigated, mainly using cell-permeable short-chain ceramide analogs, such as C-2, C-,6 or C-8 ceramide, and inhibitors that block the synthesis of ceramide (i.e., fumonisin B1) or ceramide precursor 3-ketosphinganine (i.e., myriocin). However, during influenza virus infection, the inhibition of *de novo* ceramide synthesis by inhibitor treatment was shown to affect virus replication both positively and negatively during different *in vitro* experiments (25–27). These results suggest that the ceramide pathway is important for virus replication or host defense in diverse ways. Opposing results could potentially be explained by the complexity of the sphingolipid metabolic pathway and the use of an *in vitro* experimental condition as the complete inhibition of ceramide synthesis may lead to the inhibition of other metabolites, including sphingomyelin, over time. Further, the treatment of cells with C-6 ceramide analog, which could increase many ceramide species in cells and change the lipid components of membranes, suppressed influenza virus replication and suggests an antiviral role of ceramides. On the other hand, local administration of C-8 ceramide in mice did not affect influenza virus propagation *in vivo* but increased dendritic cell stimulation and virus-specific T cell responses during infection (46). While the *de novo* sphingolipid synthesis pathway could be critical for regulating influenza virus infection, completely blocking the early stage of sphingolipid synthesis may result in diverse outcomes based on prior inhibitor studies. Currently, there are no studies investigating the role of CerS enzymes during influenza infection using genetic modification approaches. As the *in vitro* overexpression of CerS4, but not CerS1, inhibited influenza virus replication (Fig. 1), it is possible that specific ceramide subspecies synthesized in cells may be critical for the host defense against virus infection. Therefore, interrogating the role of individual CerSs during influenza virus infection could increase the comprehensive understanding of the ceramide network in explicitly regulating influenza virus replication.

CerS4 was shown to display antiviral activity against the replication of multiple strains (WSN and pH1N1) and subtypes (H1N1 and H3N2) of influenza A virus as well as influenza B virus. CerS4 activity is unaffected by the well-known CerS inhibitor fumonisin B1 (28). The function of CerS4 during infection with other viruses has not yet been reported to the best of our knowledge. Thus, it would be interesting to determine if CerS4 has similar or different roles during other virus infections for a deeper understanding of ceramide/CerS4-virus interactions.  

Influenza virus has been reported to induce degradation of many host proteins (47–52). The phenomena have been linked to influenza viral evasion or nullification of the host defense to favor virus propagation. However, the systemic and detailed pathways for influenza viral mechanisms to induce degradation of host antiviral proteins remain poorly understood (53). In this study, the antiviral host factor CerS4 was shown to be degraded during influenza virus infection, where influenza virus most likely utilizes additional host factors to cause CerS4 ubiquitination and subsequent degradation. Thus, probing the mode of action by which influenza virus induces degradation of CerS4 may help identify host pro-influenza proteins and facilitate the design of host-directed antiviral therapeutics against influenza.

## MATERIALS AND METHODS

### Viruses and cells

The influenza viruses A/WSN/33 (H1N1) and pandemic A/CA/04/09 (H1N1) were utilized as described in prior studies (54, 55). Both the A/Hong Kong/8/68 (H3N2) virus (ATCC VR-1679) and the B/Lee/40 (IBV) virus (ATCC VR-1535) were purchased through ATCC. The viruses were propagated in Madin-Darby canine kidney (MDCK) cells following methods previously reported (51, 54, 55). Virus titration was conducted using a plaque assay. CerS1- or CerS4-overexpressing HEK293 cells were a kind provision from Dr. Stephen Alexander (University of Missouri-Columbia). A549, HEK293, HEK293T, and 293 FT cells were maintained in Dulbecco’s modified Eagle’s medium (DMEM; Gibco), while MDCK cells were grown in DMEM with MEM NEAA (non-essential amino acids; Invitrogen), as described earlier (51, 54, 56, 57). For the influenza A/WSN/33 (H1N1) virus, cells were incubated with 10% fetal bovine serum (FBS)-containing medium; for the influenza A/CA/04/09 (H1N1) virus, cells were incubated with FBS-free medium containing 0.3% BSA and TPCK-treated trypsin (1 µg/mL) (55). All cells were incubated at 37°C in a CO_2_ incubator, with media supplemented with 10% FBS (Sigma) and penicillin-streptomycin (100 U/mL and 100  µg/mL, respectively; Invitrogen).

### Reagents and antibodies

Bafilomycin A1 was sourced from Cayman Chemical; MG132 and NH4Cl were purchased from Fisher Scientific. Thapsigargin was purchased from Tocris Bioscience (Bristol, UK). Anti-CerS4 antibodies were purchased from Origene (Rockville, MD). Antibodies against IAV HA were purchased from Santa Cruz Biotechnology (Dallas, TX). Antibodies specific to vial NS1 and PB1 were purchased from GeneTex (Irvine, CA). Antibodies targeting IAV M1, IAV M2, and IBV NP were purchased from Abcam (United Kingdom, Cambridge). Antibodies for p-JNK, total JNK, p-MEK, p-NFκB, p-p38, p-STAT1, HA tag, Myc tag, and GAPDH were purchased from Cell Signaling Technology (Danvers, MA).

### Plasmid constructs and transfection

The plasmid DNAs encoding human CerS1 and human CerS4, tagged with Myc and Flag, were purchased from Origene, and the pCMV-Flag plasmid was provided by Dr. David Pintel (University of Missouri-Columbia). Cultured cells were plated in 24-well plates at densities of 1 × 10⁵ cells/well at 18 hours prior to transfection. Cells were transiently transfected with plasmids encoding Myc and Flag-tagged CerS4 at a concentration of 500 ng/mL using TurboFect transfection reagent (Thermo Scientific), following the manufacturer’s recommended protocols. In all transfection experiments, pCMV-Flag (Empty vector) was used as controls to ensure that the total amount of DNA was consistent across all samples.

### Western blot analysis

Western blotting was conducted as described in previous studies (51, 54, 56, 57). In summary, denatured polypeptides from cell lysates or immunoprecipitation (IP) samples were resolved by SDS-PAGE and transferred onto nitrocellulose membranes (Bio-Rad). Membrane-bound primary antibodies were detected using HRP-conjugated secondary antibodies (Cell Signaling Technology). The signals were visualized using an Odyssey Fc imaging system (Li-Cor) and processed with Image Studio software (Li-Cor). At least two independent experiments were performed, which produced comparable results.

### Denatured immunoprecipitation assay

Detection of CerS4 ubiquitination during IAV infection was performed using A549 cells (1 × 10⁶) that were mock-infected or infected with IAV at an MOI of 2 for 24 hours. The cells were lysed with 200 µL of 1% SDS-containing lysis buffer and denatured by boiling at 95°C for 5 minutes. This process inhibits cellular ubiquitin hydrolases, preserving Ub-CerS4 conjugates, but disrupts protein-protein interactions. For IP, lysates were diluted fivefold (final volume: 1 mL) in IP lysis buffer supplemented with 1 mM PMSF and incubated by vortexing overnight at 4°C with Protein-G agarose beads (30 µL) preloaded with anti-CerS4 antibodies (Origene). After four rigorous washes to remove nonspecific interactions, the precipitated proteins were analyzed via Western blotting. The experiments were performed twice independently, with similar results. To analyze bound proteins, 20 µL of 2 × sample buffer was added to the beads for elution, followed by immunoblotting with anti-ubiquitin antibodies (Cell Signaling Technology).

### Knockdown by siRNA

Silencer Select siRNA for LASS4 (CerS4) and Silencer Select Negative Control #1 (control scrambled RNA; SCR) were purchased from Thermo Scientific. HEK293T or A549 cells were transfected with 20 nM siRNA using Lipofectamine RNAiMax transfection reagent (Thermo Scientific) according to the manufacturer’s instructions.

### Knockdown by lentivirus-derived shRNA

Lentiviral vectors pGFP-CerS4-shLenti #1 and #2 were purchased from Origene (Rockville, MD). Oligonucleotides encoding different shRNAs were cloned between the U6 promoter and the SV40 promoter. For lentivirus production, 293 FT cells were transfected with pGFP-CerS4-shLenti #1 or #2 along with packaging plasmids (PAX and MD, provided by Dr. Julie Saba, UCSF). Culture supernatants were collected 72 hours post-transfection, centrifuged at 1,500 rpm for 10 minutes at 4°C, and filtered through a 0.45 µm filter. For lentiviral transduction of A549 cells, 1 × 10^4^ cells per well were seeded in 24-well plates. After overnight incubation, 0.5 mL of culture supernatants containing sh-CerS4-#1 or a mixture of sh-CerS4 lentiviruses (pGFP-CerS4-shLenti #1 and #2) was added to the wells with 8 µg/mL polybrene (Sigma-Aldrich). The cells were incubated overnight at 37°C and then utilized for subsequent experiments or analyses. After 48 hours of transduction, A549 cells were infected with either mock or IAV pH1N1 at an MOI of 0.1 for 24 hours.

### Real-time PCR

Total cellular RNA was extracted using the DNA-free RNA Isolation Kit (Invitrogen) according to the manufacturer’s protocol. The purified RNA was reverse-transcribed into cDNA using random primers (Invitrogen) and reverse transcriptase (Promega) for detection of CerS4 and GAPDH, while primers complementary to viral (−) strand RNA were used for detection of viral (−) strand RNAs of NP or NS1. Using the cDNAs, a separate real-time quantitative PCR (RT-qPCR) was performed with gene-specific primers (56). Targets included human CerS4 (5′- CCC GAC TGG TCC TCT TTC CC −3′ and 5′-GCA GCA ACA TCA GAA GCC CG −3′), viral NP (5′- TAT GTG GCA TCA TTC AGG TTG GA-3′ and 5′- GAC GGA AAG TGG ATG AGA GAA CT −3′), viral NS1 (5′- CTC TGT CGC TTT CAA TCT GTG C-3′ and 5′-TCG CTT GGA GAA ACT GTG ATG A-3′), and human GAPDH (5′-AGC CTC AAG ATC ATC AGC AAT GCC-3′ and 5′-TGT GGT CAT GAG TCC TTC CAC GAT-3′). The qPCRs utilized Power SYBR Green PCR Master Mix (Applied Biosystems) on a StepOne real-time PCR system (Applied Biosystems), with cDNA levels normalized to GAPDH RNA in the same samples.

### Plaque assays

A549 cells were infected with IAV at the specified MOI. At the indicated time points, the supernatants were collected, and virus titers were determined using MDCK cells. For the titration of pH1N1, MDCK cell monolayers were washed with PBS supplemented with 0.3% bovine serum albumin (BSA) and subsequently infected with tenfold serial dilutions of the virus. The infected MDCK cells were overlaid with a mixture containing 0.6% SeaKem LE agarose (Lonza), 2 × L15 (Invitrogen), 0.3% BSA, and 1 µg/mL TPCK-treated trypsin, followed by incubation for 2–3 days. For the titration of WSN, instead of BSA, FBS was used in the absence of trypsin. All infections were performed at 37°C in a 5% CO₂ humidified atmosphere.

### Statistical analysis

Error bars in all figures indicate the mean values ± standard deviations (SD). Significance was determined by running Student’s *t*-test (*P* < 0.05). Densitometry for Western blots was calculated using ImageJ software. Data shown are representative of at least two independent experimental replicates.

## Data Availability

Data used in preparing this article will be provided upon request to the corresponding author.

## References

[1] Naquin A, O’Halloran A, Ujamaa D, Sundaresan D, Masalovich S, Cummings CN, Noah K, Jain S, Kirley PD, Alden NB, et al.. 2024. Laboratory-confirmed influenza-associated hospitalizations among children and adults - influenza hospitalization surveillance network, United States, 2010-2023. MMWR Surveill Summ 73:1–18. doi:10.15585/mmwr.ss7706a1 PMC11537671 39471107

[2] D’Mello T, Brammer L, Blanton L, Kniss K, Smith S, Mustaquim D, Steffens C, Dhara R, Cohen J, Chaves SS, Finelli L, Bresee J, Wallis T, Xu X, Abd Elal AI, Gubareva L, Wentworth D, Villanueva J, Katz J, Jernigan D, Centers for Disease Control and Prevention (CDC). 2015. Update: influenza activity--United States, September 28, 2014-February 21, 2015. MMWR Morb Mortal Wkly Rep 64:206–212. doi:10.15585/mmwr.mm6448a4 25742380 PMC4584716

[3] Simonsen L, Clarke MJ, Schonberger LB, Arden NH, Cox NJ, Fukuda K. 1998. Pandemic versus epidemic influenza mortality: a pattern of changing age distribution. J Infect Dis 178:53–60. doi:10.1086/515616 9652423

[4] Meltzer MI, Cox NJ, Fukuda K. 1999. The economic impact of pandemic influenza in the United States: priorities for intervention. Emerg Infect Dis 5:659–671. doi:10.3201/eid0505.990507 10511522 PMC2627723

[5] Caserta LC, Frye EA, Butt SL, Laverack M, Nooruzzaman M, Covaleda LM, Thompson AC, Koscielny MP, Cronk B, Johnson A, Kleinhenz K, Edwards EE, Gomez G, Hitchener G, Martins M, Kapczynski DR, Suarez DL, Alexander Morris ER, Hensley T, Beeby JS, Lejeune M, Swinford AK, Elvinger F, Dimitrov KM, Diel DG. 2024. Spillover of highly pathogenic avian influenza H5N1 virus to dairy cattle. Nature 634:669–676. doi:10.1038/s41586-024-07849-4 39053575 PMC11485258

[6] Claas EC, Osterhaus AD, van Beek R, De Jong JC, Rimmelzwaan GF, Senne DA, Krauss S, Shortridge KF, Webster RG. 1998. Human influenza A H5N1 virus related to a highly pathogenic avian influenza virus. Lancet 351:472–477. doi:10.1016/S0140-6736(97)11212-0 9482438

[7] Cowling BJ, Jin L, Lau EHY, Liao Q, Wu P, Jiang H, Tsang TK, Zheng J, Fang VJ, Chang Z, et al.. 2013. Comparative epidemiology of human infections with avian influenza A H7N9 and H5N1 viruses in China: a population-based study of laboratory-confirmed cases. Lancet 382:129–137. doi:10.1016/S0140-6736(13)61171-X 23803488 PMC3777567

[8] Fraser C, Donnelly CA, Cauchemez S, Hanage WP, Van Kerkhove MD, Hollingsworth TD, Griffin J, Baggaley RF, Jenkins HE, Lyons EJ, et al.. 2009. Pandemic potential of a strain of influenza A (H1N1): early findings. Science 324:1557–1561. doi:10.1126/science.1176062 19433588 PMC3735127

[9] Wang TT, Palese P. 2009. Unraveling the mystery of swine influenza virus. Cell 137:983–985. doi:10.1016/j.cell.2009.05.032 19524497

[10] Cheng PKC, To APC, Leung TWC, Leung PCK, Lee CWC, Lim WWL. 2010. Oseltamivir- and amantadine-resistant influenza virus A (H1N1). Emerg Infect Dis 16:155–156. doi:10.3201/eid1601.091304 20031069 PMC2874384

[11] Dharan NJ, Gubareva LV, Meyer JJ, Okomo-Adhiambo M, McClinton RC, Marshall SA, St George K, Epperson S, Brammer L, Klimov AI, Bresee JS, Fry AM, Oseltamivir-Resistance Working Group. 2009. Infections with oseltamivir-resistant influenza A(H1N1) virus in the United States. JAMA 301:1034–1041. doi:10.1001/jama.2009.294 19255110

[12] Marjuki H, Mishin VP, Chesnokov AP, Jones J, De La Cruz JA, Sleeman K, Tamura D, Nguyen HT, Wu H-S, Chang F-Y, Liu M-T, Fry AM, Cox NJ, Villanueva JM, Davis CT, Gubareva LV. 2015. Characterization of drug-resistant influenza A(H7N9) variants isolated from an oseltamivir-treated patient in Taiwan. J Infect Dis 211:249–257. doi:10.1093/infdis/jiu447 25124927 PMC6943751

[13] Poland GA, Jacobson RM, Ovsyannikova IG. 2009. Influenza virus resistance to antiviral agents: a plea for rational use. Clin Infect Dis 48:1254–1256. doi:10.1086/598989 19323631 PMC2831648

[14] Jones JC, Kumar G, Barman S, Najera I, White SW, Webby RJ, Govorkova EA. 2018. Identification of the I38T PA substitution as a resistance marker for next-generation influenza virus endonuclease inhibitors. mBio 9:e00430-18. doi:10.1128/mBio.00430-18 29691337 PMC5915737

[15] Kumari R, Sharma SD, Kumar A, Ende Z, Mishina M, Wang Y, Falls Z, Samudrala R, Pohl J, Knight PR, Sambhara S. 2023. Antiviral approaches against influenza virus. Clin Microbiol Rev 36:e0004022. doi:10.1128/cmr.00040-22 36645300 PMC10035319

[16] Kageyama-Yahara N, Riezman H. 2006. Transmembrane topology of ceramide synthase in yeast. Biochem J 398:585–593. doi:10.1042/BJ20060697 16756512 PMC1559446

[17] Park JW, Park WJ, Futerman AH. 2014. Ceramide synthases as potential targets for therapeutic intervention in human diseases. Biochim Biophys Acta 1841:671–681. doi:10.1016/j.bbalip.2013.08.019 24021978

[18] Levy M, Futerman AH. 2010. Mammalian ceramide synthases. IUBMB Life 62:347–356. doi:10.1002/iub.319 20222015 PMC2858252

[19] Mizutani Y, Kihara A, Igarashi Y. 2005. Mammalian Lass6 and its related family members regulate synthesis of specific ceramides. Biochem J 390:263–271. doi:10.1042/BJ20050291 15823095 PMC1184580

[20] Mizutani Y, Kihara A, Igarashi Y. 2006. LASS3 (longevity assurance homologue 3) is a mainly testis-specific (dihydro)ceramide synthase with relatively broad substrate specificity. Biochem J 398:531–538. doi:10.1042/BJ20060379 16753040 PMC1559458

[21] Raichur S. 2020. Ceramide synthases are attractive drug targets for treating metabolic diseases. Front Endocrinol (Lausanne) 11:483. doi:10.3389/fendo.2020.00483 32849276 PMC7403459

[22] Li Y, Chaurasia B, Rahman MM, Kaddai V, Maschek JA, Berg JA, Wilkerson JL, Mahmassani ZS, Cox J, Wei P, et al.. 2023. Ceramides increase fatty acid utilization in intestinal progenitors to enhance stemness and increase tumor risk. Gastroenterology 165:1136–1150. doi:10.1053/j.gastro.2023.07.017 37541526 PMC10592225

[23] Hammerschmidt P, Brüning JC. 2022. Contribution of specific ceramides to obesity-associated metabolic diseases. Cell Mol Life Sci 79:395. doi:10.1007/s00018-022-04401-3 35789435 PMC9252958

[24] Raichur S, Brunner B, Bielohuby M, Hansen G, Pfenninger A, Wang B, Bruning JC, Larsen PJ, Tennagels N. 2019. The role of C16:0 ceramide in the development of obesity and type 2 diabetes: CerS6 inhibition as a novel therapeutic approach. Mol Metab 21:36–50. doi:10.1016/j.molmet.2018.12.008 30655217 PMC6407366

[25] Soudani N, Hage-Sleiman R, Karam W, Dbaibo G, Zaraket H. 2019. Ceramide suppresses influenza A virus replication in vitro. J Virol 93:e00053-19. doi:10.1128/JVI.00053-19 30700605 PMC6430560

[26] Tafesse FG, Sanyal S, Ashour J, Guimaraes CP, Hermansson M, Somerharju P, Ploegh HL. 2013. Intact sphingomyelin biosynthetic pathway is essential for intracellular transport of influenza virus glycoproteins. Proc Natl Acad Sci USA 110:6406–6411. doi:10.1073/pnas.1219909110 23576732 PMC3631694

[27] Hidari KIPJ, Suzuki Y, Suzuki T. 2006. Suppression of the biosynthesis of cellular sphingolipids results in the inhibition of the maturation of influenza virus particles in MDCK cells. Biol Pharm Bull 29:1575–1579. doi:10.1248/bpb.29.1575 16880607

[28] Riebeling C, Allegood JC, Wang E, Merrill AH, Futerman AH. 2003. Two mammalian longevity assurance gene (LAG1) family members, trh1 and trh4, regulate dihydroceramide synthesis using different fatty acyl-CoA donors. J Biol Chem 278:43452–43459. doi:10.1074/jbc.M307104200 12912983

[29] Tidhar R, Zelnik ID, Volpert G, Ben-Dor S, Kelly S, Merrill AH, Futerman AH. 2018. Eleven residues determine the acyl chain specificity of ceramide synthases. J Biol Chem 293:9912–9921. doi:10.1074/jbc.RA118.001936 29632068 PMC6016465

[30] Laviad EL, Albee L, Pankova-Kholmyansky I, Epstein S, Park H, Merrill AH, Futerman AH. 2008. Characterization of ceramide synthase 2: tissue distribution, substrate specificity, and inhibition by sphingosine 1-phosphate. J Biol Chem 283:5677–5684. doi:10.1074/jbc.M707386200 18165233

[31] Min J, Mesika A, Sivaguru M, Van Veldhoven PP, Alexander H, Futerman AH, Alexander S. 2007. (Dihydro)ceramide synthase 1–regulated sensitivity to cisplatin is associated with the activation of p38 mitogen-activated protein kinase and is abrogated by sphingosine kinase 1. Mol Cancer Res 5:801–812. doi:10.1158/1541-7786.MCR-07-0100 17699106

[32] Jęśko H, Wencel PL, Wójtowicz S, Strosznajder J, Lukiw WJ, Strosznajder RP. 2020. Fingolimod affects transcription of genes encoding enzymes of ceramide metabolism in animal model of Alzheimer’s disease. Mol Neurobiol 57:2799–2811. doi:10.1007/s12035-020-01908-3 32356173 PMC7253528

[33] Dou D, Revol R, Östbye H, Wang H, Daniels R. 2018. Influenza A virus cell entry, replication.virion assembly and movement. Front Immunol 9:1581. doi: 10.3389/fimmu.2018.01581 30079062 10.3389/fimmu.2018.01581 PMC6062596

[34] Carter T, Iqbal M. 2024. The influenza A virus replication cycle: a comprehensive review. Viruses 16:316. doi:10.3390/v16020316 38400091 PMC10892522

[35] Stith JL, Velazquez FN, Obeid LM. 2019. Advances in determining signaling mechanisms of ceramide and role in disease. J Lipid Res 60:913–918. doi:10.1194/jlr.S092874 30846529 PMC6495170

[36] Stancevic B, Kolesnick R. 2010. Ceramide‐rich platforms in transmembrane signaling. FEBS Lett 584:1728–1740. doi:10.1016/j.febslet.2010.02.026 20178791 PMC4440589

[37] Kurz J, Barthelmes J, Blum L, Ulshöfer T, Wegner MS, Ferreirós N, Roser L, Geisslinger G, Grösch S, Schiffmann S. 2019. Role of ceramide synthase 2 in G-CSF signaling and G-CSF-R translocation into detergent-resistant membranes. Sci Rep 9:747. doi:10.1038/s41598-018-37342-8 30679689 PMC6345911

[38] Zhang J, Ruan T, Sheng T, Wang J, Sun J, Wang J, Prinz RA, Peng D, Liu X, Xu X. 2019. Role of c-Jun terminal kinase (JNK) activation in influenza A virus-induced autophagy and replication. Virology (Auckl) 526:1–12. doi:10.1016/j.virol.2018.09.020 PMC6424123 30316042

[39] Nacken W, Ehrhardt C, Ludwig S. 2012. Small molecule inhibitors of the c-Jun N-terminal kinase (JNK) possess antiviral activity against highly pathogenic avian and human pandemic influenza A viruses. Biol Chem 393:525–534. doi:10.1515/hsz-2011-0270 22628315

[40] Chen CL, Lin CF, Chang WT, Huang WC, Teng CF, Lin YS. 2008. Ceramide induces p38 MAPK and JNK activation through a mechanism involving a thioredoxin-interacting protein-mediated pathway. Blood 111:4365–4374. doi:10.1182/blood-2007-08-106336 18270325

[41] Niaudet C, Bonnaud S, Guillonneau M, Gouard S, Gaugler MH, Dutoit S, Ripoche N, Dubois N, Trichet V, Corre I, Paris F. 2017. Plasma membrane reorganization links acid sphingomyelinase/ceramide to p38 MAPK pathways in endothelial cells apoptosis. Cell Signal 33:10–21. doi:10.1016/j.cellsig.2017.02.001 28179144

[42] Yue J, López JM. 2020. Understanding MAPK signaling pathways in apoptosis. Int J Mol Sci 21:2346. doi:10.3390/ijms21072346 32231094 PMC7177758

[43] Basu S, Kolesnick R. 1998. Stress signals for apoptosis: ceramide and c-Jun kinase. Oncogene 17:3277–3285. doi:10.1038/sj.onc.1202570 9916990

[44] Ip YT, Davis RJ. 1998. Signal transduction by the c-Jun N-terminal kinase (JNK) — from inflammation to development. Curr Opin Cell Biol 10:205–219. doi:10.1016/S0955-0674(98)80143-9 9561845

[45] Nacken W, Anhlan D, Hrincius ER, Mostafa A, Wolff T, Sadewasser A, Pleschka S, Ehrhardt C, Ludwig S. 2014. Activation of c-jun N-terminal kinase upon influenza A virus (IAV) infection is independent of pathogen-related receptors but dependent on amino acid sequence variations of IAV NS1. J Virol 88:8843–8852. doi:10.1128/JVI.00424-14 24872593 PMC4136289

[46] Pritzl CJ, Seo YJ, Xia C, Vijayan M, Stokes ZD, Hahm B. 2015. A ceramide analogue stimulates dendritic cells to promote T cell responses upon virus infections. J Immunol 194:4339–4349. doi:10.4049/jimmunol.1402672 25810392 PMC4402267

[47] Rodriguez A, Pérez-González A, Nieto A. 2007. Influenza virus infection causes specific degradation of the largest subunit of cellular RNA polymerase II. J Virol 81:5315–5324. doi:10.1128/JVI.02129-06 17344288 PMC1900203

[48] Wang S, Chi X, Wei H, Chen Y, Chen Z, Huang S, Chen JL. 2014. Influenza A virus-induced degradation of eukaryotic translation initiation factor 4B contributes to viral replication by suppressing IFITM3 protein expression. J Virol 88:8375–8385. doi:10.1128/JVI.00126-14 24829357 PMC4135930

[49] Jung KI, Ko DH, Shin N, Pyo CW, Choi SY. 2019. Endoplasmic reticulum-associated degradation potentiates the infectivity of influenza A virus by regulating the host redox state. Free Radic Biol Med 135:293–305. doi:10.1016/j.freeradbiomed.2019.03.021 30905731

[50] Jung KI, Pyo CW, Choi SY. 2018. Influenza A virus-induced autophagy contributes to enhancement of virus infectivity by SOD1 downregulation in alveolar epithelial cells. Biochem Biophys Res Commun 498:960–966. doi:10.1016/j.bbrc.2018.03.089 29548827

[51] Xia C, Vijayan M, Pritzl CJ, Fuchs SY, McDermott AB, Hahm B. 2015. Hemagglutinin of influenza A virus antagonizes Type I Interferon (IFN) responses by inducing degradation of Type I IFN receptor 1. J Virol 90:2403–2417. doi:10.1128/JVI.02749-15 26676772 PMC4810695

[52] Wolf JJ, Xia C, Studstill CJ, Ngo H, Brody SL, Anderson PE, Hahm B. 2021. Influenza A virus NS1 induces degradation of sphingosine 1-phosphate lyase to obstruct the host innate immune response. Virology (Auckl) 558:67–75. doi:10.1016/j.virol.2021.02.006 PMC8109848 33730651

[53] Xia C, Wang T, Hahm B. 2024. Triggering degradation of host cellular proteins for robust propagation of influenza viruses. Int J Mol Sci 25:4677. doi:10.3390/ijms25094677 38731896 PMC11083682

[54] Xia C, Seo YJ, Studstill CJ, Vijayan M, Wolf JJ, Hahm B. 2018. Transient inhibition of sphingosine kinases confers protection to influenza A virus infected mice. Antiviral Res 158:171–177. doi:10.1016/j.antiviral.2018.08.010 30125617 PMC6190705

[55] Xia C, Wolf JJ, Vijayan M, Studstill CJ, Ma W, Hahm B. 2018. Casein kinase 1α mediates the degradation of receptors for Type I and Type II interferons caused by hemagglutinin of influenza A virus. J Virol 92:e00006-18. doi:10.1128/JVI.00006-18 29343571 PMC5972889

[56] Seo YJ, Blake C, Alexander S, Hahm B. 2010. Sphingosine 1-phosphate-metabolizing enzymes control influenza virus propagation and viral cytopathogenicity. J Virol 84:8124–8131. doi:10.1128/JVI.00510-10 20519401 PMC2916542

[57] Vijayan M, Seo YJ, Pritzl CJ, Squires SA, Alexander S, Hahm B. 2014. Sphingosine kinase 1 regulates measles virus replication. Virology (Auckl) 450–451:55–63. doi:10.1016/j.virol.2013.11.039 PMC3918136 24503067

